# Development and Validation of Methods for the Determination of Anthocyanins in Physiological Fluids via UHPLC-MS^n^

**DOI:** 10.3390/molecules25030518

**Published:** 2020-01-24

**Authors:** Michael Kaiser, Lisa Müller-Ehl, Maike Passon, Andreas Schieber

**Affiliations:** Department of Nutritional and Food Sciences, Molecular Food Technology, University of Bonn, Endenicher Allee 19b, 53115 Bonn, Germany

**Keywords:** anthocyanins, anthocyanidins, metabolites, urine, plasma, proteolysis, LC-MS analysis

## Abstract

Recent in vitro and in vivo studies on anthocyanins confirmed numerous health-promoting effects in humans. Daily anthocyanin intake can be estimated via food databases, but the amount absorbed by the organism still remains uncertain because anthocyanin bioavailability is yet to be elucidated in its entirety. For this purpose, suitable and validated methods of sample preparation and analysis are required. Therefore, a sample preparation method for anthocyanin metabolite analysis in plasma was successfully established and validated. The validation yielded acceptable results for the anthocyanins in terms of recovery (54–108%) and precision (coefficient of variation (CV) < 15%). The UHPLC-MS method used in the consecutive reaction monitoring (CRM) mode was sufficiently sensitive, resulting in limits of detection <2.3 ng/mL and limits of quantification < 8.1 ng/mL with associated repeatability of the MS system with CVs of <5.1%. In addition, a method for the sum parameter determination of anthocyanidins in urine comprising solely the evaporation of acidified samples was developed, validated, and successfully applied to real samples. The results showed that this method is applicable for the methylated anthocyanidins, but not for the hydroxylated anthocyanidins, due to the chosen CRM modes required for optimum selectivity.

## 1. Introduction

Anthocyanins form the largest and most important class of water-soluble plant pigments [1]. They are responsible for the blue, violet, and red colors of many botanical tissues. In recent years, the bioavailability of anthocyanins gained increasing interest because of their attributed health benefits [2]. In vitro studies confirmed antioxidative, antimicrobial, anti-inflammatory, anti-obesity, and antidiabetic effects [3], with the latter being caused by decreasing both the Na^+^-dependent and -independent glucose uptake in intestinal epithelial cells [4]. Following further indications from epidemiological studies, anticancer effects for individual anthocyanins and anthocyanin mixtures from fruit or vegetable extracts were also demonstrated in vitro [4]. For example, a blueberry anthocyanin extract, similar in its composition to bilberry [5], caused increased apoptosis in human colon cancer HT-29 and in mouse melanoma B16-F10 cells. Likewise, different effects of anthocyanins and their phase II metabolites were recently demonstrated and discussed with respect to several types of cancer [4]. Accordingly, positive effects on the cardiovascular system and protection against neurodegenerative diseases like Alzheimer’s disease and dementia, via suppressing dopaminergic cell death by interfering with the microglial activation and amelioration of mitochondrial dysfunction, were reported [3,4]. In vivo, most of these results and further neuroprotective effects were verified [3,6]. In addition, positive effects on visual health were discussed [7,8]. However, the particular mode of action and the effective dose are yet to be clarified regarding these health-promoting effects [3,4]. At least some effects, like the antioxidative and anti-inflammatory properties, are structure-dependent, specifically the substitution pattern of the anthocyanin B-ring. The solely hydroxylated delphinidin (Dp) and cyanidin (Cy) derivatives are, amongst others, effective in suppressing inflammatory peritonitis by inhibiting the expression of cyclooxygenase-2, whereas methoxylated anthocyanins like peonidin (Pn), petunidin (Pt), and malvidin (Mn) derivatives showed no effect on cyclooxygenase expression [4]. Since the availability of human target tissues is very limited, the determination of the concentration in the organism and bioavailability in total is primarily based on the measurements of metabolites in plasma or urine [2]. Recently, the analysis of low-molecular-weight phenolic acids, produced by the action of the colonic microbiota, gained importance; thus, feces samples were collected during several human studies and analyzed [9,10], as these products appeared to be the main metabolites of the anthocyanins occurring in the human organism [11,12]. Although these phenolic acids cause some beneficial effects on human health, the majority of the proven and discussed health-promoting effects are directly linked to anthocyanins and their phase II metabolites [3,4]. As mentioned before, both the concentration and the structure of the anthocyanins and their metabolites present in the organism are crucial for the understanding of their physiological effects [13].

The anthocyanidin structure is pH-dependent, and the structural transformations are in chemical equilibrium. Thus, at pH 1–3, the anthocyanins are mainly present as red flavylium cations. When the pH rises to 4–5, the equilibrium is shifted to the colorless carbinol base (a pseudo base). The violet quinoid base is formed via proton transfer and occurs largely at a pH range of 6–7; it is further deprotonated at pH 7–8 to a blue ionic anhydrous base, from which a yellow chalcone emerges due to ring opening. At pH > 8, the chalcone rearranges mostly to an unstable α-diketone, which immediately decomposes to the corresponding phenolic acid and phloroglucinol aldehyde [14,15]. Accordingly, the anthocyanins are not present in the plasma or urine as flavylium cations but, pursuant to the particular pH [16,17], mostly as their quinoid base in plasma (pH 6.8) or as their carbinolic and quinoic base in urine (pH 5.5–6.5). Prior to UHPLC-MS analysis, anthocyanidins should be present as flavylium cations to enable sufficient chromatographic separation and efficient ionization.

In the organism, anthocyanins were found as glycosides, glucuronides, sulfates, methylated conjugates, and mixed conjugates after passing the hepatic phase II metabolism and the enterohepatic circulation [18]. In plasma, anthocyanins and their metabolites are mostly bound to proteins, particularly to human serum albumin (HSA) [18], the main plasma protein [16]. In addition, globulins and bioactive proteins such as enzymes and peptide hormones are present [19]. The decomposition of the primary structure of the proteins leads to the release of non-covalently bound metabolites and makes them available for extraction. Therefore, the innovative method using plasma proteolysis established to analyze quercetin-3-*O*-glucuronide [20] was adapted for the analysis of anthocyanin derivatives. Previously published and validated methods used conventional techniques like solid-phase extraction (SPE) or protein precipitation, often combined with organic liquid-liquid extraction (LLE) [21,22,23,24,25]. Plasma is a challenging matrix because of its complex composition [16]. Urine, on the other hand, is composed of approximately 95% water, with dissolved urea, creatinine, uric acid, and electrolytes being the main components. In contrast to plasma, it contains a negligible amount of proteins [26]. Therefore, it is a less challenging matrix compared to plasma and requires less sample preparation, like evaporation [27], or solely membrane filtration [28]. Yet, the most common sample preparation method regarding the analysis of anthocyanins in urine samples seems to be SPE [9,22].

**Figure 1:**
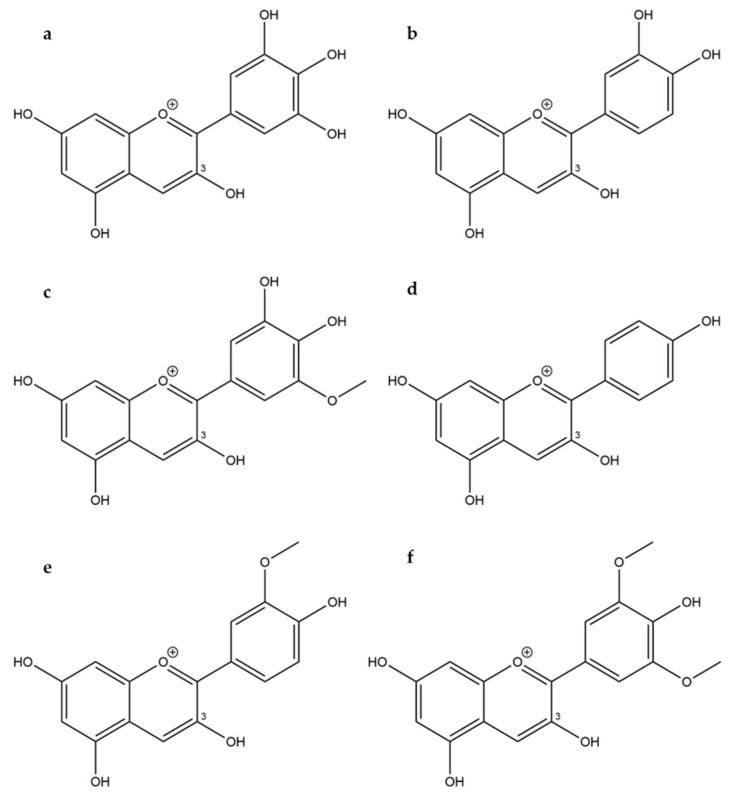
Structures of (**a**) delphinidin, (**b**) cyanidin, (**c**) petunidin, (**d**) pelargonidin, (**e**) peonidin, and (**f**) malvidin.

The six most common anthocyanidins are Cy, Dp, Mn, pelargonidin (Pg), Pn, and Pt (Figure 1) [14]. Bilberries (*Vaccinium myrtillus* L.) are anthocyanin-rich fruits with a total anthocyanin content ranging from 290 to 450 mg/100 g fresh weight [29], including over 15 anthocyanins [5]. Therefore, these fruits and also blueberries were frequently used in anthocyanin bioavailability studies [21,30,31]. All major anthocyanidins, except for Pg, occur in bilberries as their respective 3-*O*-glucosides (glc), 3-*O*-galactosides (gal), or 3-*O*-arabinosides (ara) [29]. Since Pg and its derivatives cannot be metabolized from the present anthocyanins [18], they constitute suitable internal standards for method development and validation.

In this study, an innovative method to prepare plasma samples using enzymatic proteolysis and an easy-to-handle method for the preparation of urine samples deriving from human intervention studies were comprehensively validated, with bilberry-derived anthocyanins [29]. Bilberry was chosen because all major anthocyanidin B-ring substituents are present [32].

## 2. Results and Discussion

### 2.1. Anthocyanidins in Urine

In human intervention studies, urine samples are often collected to monitor the metabolism and the excretion of polyphenolic compounds. The enzymatic release of the phase II metabolites by sulfatases and glucuronidases should be interpreted with caution because of the well-known disadvantages [33]. However, enzymatic hydrolysis is often needed to increase the concentration of the aglycones and to ensure their accurate quantification, as anthocyanidin reference compounds are commercially available in contrast to their phase II metabolites [34]. Currently, there is still a lack of methods which include the comprehensive validation of anthocyanidin analysis in urine.

**Figure 2:**
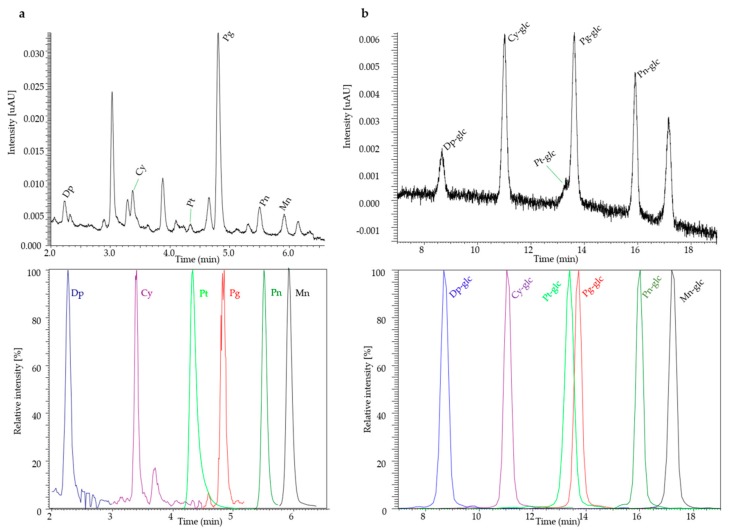
Chromatographic profiles (top: UV at 520 nm; bottom: extracted ion chromatograms) of (**a**) anthocyanidins in urine and (**b**) anthocyanins in plasma. (Dp (*m*/*z*) 303→257→229; Cy (*m*/*z*) 287→213; Pt (*m*/*z*) 317→302→274; Pg *(m*/*z*) 271→149 and 215; Pn (*m*/*z*) 301→286→258; Mn (*m*/*z*) 331→316→299; Dp-glc (*m*/*z*) 465→303; Cy-glc (*m*/*z*) 449→287; Pt-glc (*m*/*z*) 479→317; Pg-glc (*m*/*z*) 433→271; Pn-glc (*m*/*z*) 463→301; Mn-glc (*m*/*z*) 493→331).

The results for the validation of the anthocyanidins are shown in Table 1. It is obvious that the analytical limits for the hydroxy-substituted anthocyanidins, Dp, Cy, and Pg, differ significantly from those of Pn, Pt, and Mn, which are methoxy-substituted agylcones. Concerning the hydroxylated anthocyanidins, the limit of detection (LOD) ranged from approximately 60–125 ng/mL and the limit of quantification (LOQ) ranged from 200–430 ng/mL, whereas the ranges for the methoxy-substituted were between 5–13 ng/mL and 19–46 ng/mL, respectively. The lower LOQs (LLOQs) are estimated as the lower limit of the confidence interval of the LOQ and describe the lower limit that is statistically admissible for quantification [35]. The analytical limits of the methoxylated anthocyanidins are within an acceptable range [21,24,28]. Accordingly, the values for the linearity of the hydroxylated anthocyanins are 25-fold higher than those of the methoxy-substituted ones. The linearity showed a correlation of *r²* > 0.997 for each analyte. The parameters LOD, LOQ, LLOQ, and the linearity directly depend on the performance specifications of the instrument, as well as on the fragmentation settings. Because, under the extraction conditions employed, anthocyanidins are present as the flavylium cation, the ionizability should not be influenced and may not explain these differences. Although interfering matrix compounds may affect the ionization efficacy [36], it is likely that the fragmentation settings exert a greater effect. However, the actual influence of the matrix effect was not determined as it is not mandatory regarding either the validation guideline used [35] or the Food and Drug Administration (FDA) guidelines [37,38]. The energy used for collision-induced dissociation affected the formation and the intensity of the product ions. To achieve the transitions described in Table 2, the ring structures of Dp, Cy, and Pg need to be cleaved, which requires a high energy, which led to several different fragments of lower intensity. In contrast, the loss of the methoxy group necessitates a lower energy, which resulted in one product ion with a high intensity. Yet, the advantages of an improved selectivity of the consecutive reaction monitoring (CRM) mode applied outweigh the disadvantages of the diminished sensitivity, as a high selectivity is mandatory to avoid false negative results [39]. Furthermore, the linear ion trap used has two conversion dynodes, and, when operated in CRM mode, the sensitivity is only slightly decreased compared to a standard triple quadrupole operated in multiple reaction monitoring (MRM) mode. Figure 2a shows the high selectivity of the MS/MS conditions applied compared to the UV chromatogram at 520 nm. The anthocyanins of interest are distinguishable by their *m*/*z* ratio; however, in physiological samples, despite matrix separation, there are still interfering compounds that have the same *m*/*z* values. With three or more stages of *m*/*z* separation in CRM mode, the selectivity is increased, albeit at the expense of sensitivity. For the methoxy-substituted anthocyanins, the repeatability of the UHPLC-MS^n^ system was excellent with a coefficient of variation (CV) < 5%, and, for the hydroxy-substituted aglycones, it was acceptable with a CV < 10% [39]. The CVs of the method precisions of Pt, Pn, and Mn were < 20% and < 30% for Cy. These results are in an acceptable range for physiological samples [39] and in accordance with the validation guidelines [35].

For the methoxy-substituted anthocyanins, the repeatability of the UHPLC-MS^n^ system was excellent with a coefficient of variation (CV) < 5%, and, for the hydroxy-substituted aglycones, it was acceptable with a CV < 10% [39]. The CVs of the method precisions of Pt, Pn, and Mn were < 20% and < 30% for Cy. These results are in an acceptable range for physiological samples [39] and in accordance with the validation guidelines [35]. However, the FDA regulations recommended CVs < 15% [37]. Due to the high analytical limits and the high CV values of the method precision, the established method is less suitable for the analysis of solely hydroxylated anthocyanidins like Cy and Dp. Accordingly, the method presented here shows good suitability for the analysis of methylated anthocyanidins with satisfactory recoveries of about > 65%. This is of particular importance because methylation is part of the hepatic phase II metabolism [18], and the enzymatic release of these metabolites is not possible [33]. For a comprehensive analysis, methylated metabolites need to be determined in addition to the aglycones, which is possible with the established method. The high CV of the method precision shows strong systemic biases, which may be reduced by a higher grade of automation [40]. However, an entire automation of the method is difficult or even impractical because of its design. Only the use of a pipetting robot during sample preparation seems possible to avoid systemic biases caused by the human factor. Therefore, the CV of the method precision might be similarly diminished for all analytes [40]. However, due to the comparatively high CVs, a reduction of systemic biases via automation is desirable.

The sample preparation technique using evaporation of urine for the analysis of anthocyanins was first proposed by Netzel and co-workers [27]. In this study, this procedure was adjusted to the less stable anthocyanidins. This could already be achieved by increasing the amount of formic acid added before evaporation. Previous methods were mostly validated for anthocyanin glucosides as substitutes for their phase II metabolites but not for their aglycones [9,22]. In recent years, human urine samples from nutritional studies were often analyzed using methods for which no validation data could be found [21,31,41] or which were insufficiently validated, e.g., only the recovery [42] or the recovery of the internal standard was determined [43,44]. To evaluate the results of these studies, it is mandatory to have information on all validation parameters. Otherwise, the suitability and the quality of the methods remain uncertain, and the results might at least be challenged [39].

Although SPE combines the advantages of cleanup and concentration, the poor batch-to-batch reproducibility is a frequently occurring problem. Furthermore, the possible leaching of solid-phase material impacts the MS response. These issues of SPE are avoided with the method presented here. The addition of formic acid leads to precipitation of the proteins, which are removed during membrane filtration prior to analysis, as they would be via SPE. These benefits outweigh the comparatively lower degree of possible automation. In contrast to the previously published and conventionally applied methods using SPE [9,22,41,42,43,44], this extensively validated method allows a valid quantification of anthocyanidins in human urine. The omission of SPE results in low material costs and contributes to waste minimization. This and the exclusion of organic solvents contribute to greater environmental compatibility.

The application of this sample preparation method was tested with some residual urine samples originating from a human intervention study conducted in 2017 at the Department of Nutritional and Food Sciences of the University of Bonn. As expected, the determination of the methylated anthocyanidins was satisfactory (Table 3). Anthocyanin phase II metabolites were released with sulfatase and β-glucuronidase prior to evaporation. The measured concentrations of Pt, Pn, and Mn were above the respective LLOQ (Table 1) and were, therefore, quantifiable in the quadruplicate analysis of each sample. In contrast, the hydroxylated anthocyanins Dp and Cy were either not detectable or not quantifiable due to the fact that the concentration was below the analytical limits. The data shown in Table 3 yielded lower CVs regarding the precision than listed in Table 1. The averaged CVs of the measured concentrations were 10.2% for Pt, 6.8% for Pn, and 9.4% for Mn, and, thus, below the acceptance limit of 15% as required by the FDA [37], even without further automation as discussed earlier. Accordingly, the method proved to be suitable, with the limitations mentioned previously regarding solely hydroxylated anthocyanidins, for the sum parameter analysis of methoxylated anthocyanidins in real human urine samples.

### 2.2. Anthocyanins in Plasma

In plasma, the analysis of anthocyanin phase II metabolites rather than the aglycones is the focus of current research [22,23,24,25]. Because reference compounds of anthocyanin phase II metabolites are not commercially available [34], the method was developed and validated using anthocyanin glucosides. It is a common procedure to quantify these phase II metabolites by the substitutional use of their commercially available respective glucosides [20,34]. Figure 2b shows the high selectivity of the MS/MS conditions applied and a UV chromatogram at 520 nm representing a sample with an anthocyanin concentration of 5 µg/mL. Samples containing lower concentrations did not provide any usable UV signals. The calibration curves of the anthocyanins were in general linear from 2 ng/mL–25 µg/mL with a coefficient of determination *r²* of > 0.999. The individual linearity ranges of the different glucosides and all further validation parameters are listed in Table 4. These were broader than those previously reported for anthocyanins, as the values ranged between 0.1 ng/mL and 200 ng/mL [22,23] or from 0.5–9 µg/mL [24]. Furthermore, the coefficient of determination *r²* was in agreement [23] or even better [22,24,25], even though a matrix-matched calibration without SPE was performed. Plasma samples hydrolyzed with protease were used as solvent as described earlier [20]. The method presented here was less sensitive, as the LOD of 1–2 ng/mL was about 10-fold higher, and the LOQ of 4–8 ng/mL was more than 20-fold higher than those previously reported [22,23]. However, this lower sensitivity, on the one hand, can be considered device-dependent, as triple quadrupole (TQD) MS systems equipped with either a turbo ion spray (TIS) interface [22] or an electrospray ionization (ESI) interface [23] were used instead of the linear ion trap. On the other hand, the higher matrix load compared to the SPE eluates might have caused the reduced sensitivity due to ion suppression and other matrix effects [36]. The validation data demonstrated that the MS system used can reproducibly detect the analytes and the internal standard with high precision because the CVs of the repeatability of the measuring instrument were ≤ 5% [39]. Nonetheless, the linearity range and the sensitivity cover the working range necessary for plasma samples derived from nutritional studies, as the reported concentrations were from 1 ng/mL to 135 ng/mL [21,24,31].

The intra-day and inter-day method precisions with CVs of < 15.5% can be considered within the acceptable range of physiological samples stated by the FDA. The method presented here is more precise than the method described by Liu and co-workers, who reported a CV up to 18% [25]. All other aforementioned methods show corresponding CVs [22,23,24]. The recoveries ranging from 54% for Dp-glc up to 108% for Mn-glc were in accordance with the applied validation guidelines [35], and they were comparable to those stated for the other validated methods [22,23,24,25]. The established method provided validation data for the glucosides of five of the six major anthocyanidins, which enables comprehensive analysis of all types of anthocyanin derivatives, whereas other studies mostly validated the methods using only Dp or Cy derivatives [22,23,25]. It is noticeable that, in each method, the lowest recovery was determined for Dp derivatives, which Nakamura and co-workers attributed to the lower chemical stability compared to other anthocyanidin derivatives [23]. Hydroxylated anthocyanidins are known to form hydrogen bonds or even complexes with metal ions [45] circulating in the plasma. Methoxy groups are far less reactive toward plasma components than hydroxyl groups [45,46], which is why the glucosides of Mn and Pn show the best recoveries. Because the samples were stable for at least 16 h at 10 °C, sample preparation can be performed during the day, and UHPLC-MS analysis can take place overnight.

The previously developed method for the analysis of quercetin derivatives using pepsin-induced plasma proteolysis as a step of sample preparation [20] was successfully expanded to the analysis of anthocyanin derivatives without any changes. It is, therefore, assumed that other substance classes such as phenolic acids may also be analyzed using this method. The validation data showed better results for the anthocyanins analysis in terms of method precision, recovery, and repeatability than for the analysis of quercetin derivatives [20]. Thus, another alternative to the common sample preparation techniques, such as SPE, was developed and established to analyze anthocyanin derivatives in plasma samples. Compared to conventional SPE methods, the required material and the resulting waste were significantly reduced. With the method presented here, the previously discussed disadvantages of SPE, such as the poor batch-to-batch reproducibility, are avoided and the cleanup and concentration of the analytes is still ensured. Furthermore, plasma may plug the SPE columns, which would entail the loss of the sample for analysis. The simultaneous analysis of phase II metabolites of anthocyanins and their different polarity, as sulfates are more polar than glucuronides [20], is quite challenging during SPE. This problem does not play a role in the method presented here. Moreover, a complete release of the protein-bound anthocyanins can be achieved through the comprehensive proteolysis, which is not necessarily guaranteed by the conventional protein precipitation [20]. Unfortunately, no plasma samples derived from the human intervention study were available to test the application under these conditions. Nevertheless, the validation data obtained are promising. In the future, even an adaptation of the method for the sum parameter determination of anthocyanin metabolites as anthocyanidins seems conceivable. For this purpose, enzymatic hydrolysis of the metabolites via sulfatase and β-glucuronidase, as described before, has to be executed after proteolysis and prior to organic precipitation.

Automation of this method to improve the method precision by reducing the systemic biases [40] appears to be difficult as it consists of many different steps. Only the use of a pipetting robot during sample preparation might be feasible. However, in view of the low CVs regarding the method precision, further automation of the method is not mandatory.

## 3. Materials and Methods

### 3.1. Chemicals and Reagents

Pepsin from porcine mucosa (63 units/mg), sulfatase type VI from *Aerobacter aerogenes* (2–5 units/mg protein), β-glucuronidase from *Escherichia coli* (≥20 000 units/mg protein), and reagent-grade formic acid were purchased from Sigma-Aldrich (Steinheim, Germany). Water was purified by a Purelab Flex Water Purification System from ELGA LabWater (High Wycombe, UK). HPLC-grade methanol was obtained from Th. Geyer (Renningen, Germany). Acetic acid (100%) was purchased from VWR International (Darmstadt, Germany). Cyanidin-3-*O*-glucoside chloride, delphinidin chloride, malvidin-3-*O*-glucoside chloride, pelargonidin-3-*O*-glucoside chloride, and peonidin-3-*O*-glucoside chloride, with a purity of >97% each, were obtained from PhytoPlan Diehm & Neuberger GmbH (Heidelberg, Germany). Petunidin-3-*O*-glucoside chloride (>98%) was purchased from Polyphenols Laboratories AS (Sandnes, Norway). Delphinidin-3-*O*-glucoside chloride (>96%), as well as cyanidin chloride, malvidin chloride, pelargonidin chloride, and peonidin chloride, with a purity of >97% each, was acquired from Extrasynthese (Genay, France). Petunidin chloride (>97%) was purchased from Cayman Chemical (Ann Arbor, MI, USA). Acetonitrile and water, both LC-MS grade, were obtained from Th. Geyer (Renningen, Germany), and formic acid was purchased from Honeywell (Seelze, Germany). Human plasma was provided by the University Hospital Bonn, Germany. Anthocyanin-free urine was obtained from volunteers (male, age 30–35) after three-day abstinence of anthocyanin-containing food.

### Stock Solutions

Stock solutions of the anthocyanins were prepared in methanol/water/acetic acid 70:29:1 (*v*/*v*), and the anthocyanidins were dissolved in methanol/water/formic acid 70:25:5 (*v*/*v*). With the exception of the internal standard pelargonidin, all anthocyanins and all anthocyanidins were combined in a stock solution (200 µg/mL). The stock solution of pepsin (400 ng/mL) was prepared in 1% (*v*/*v*) aqueous formic acid. The concentration of the stock solutions of the internal standards was 200 µg/mL each.

### 3.2. Sample Preparation

#### 3.2.1. Urine

The urine samples were obtained from an intervention study. All procedures involving human subjects and study protocols were approved and accepted by the ethics commission of the Medical Faculty of the University of Bonn (project identification code 019/17) and performed in accordance with the Declaration of Helsinki. The intervention study was part of the project Diet Body Brain (BMBF, Grant No: 01EA1410A). The study was conducted in 2017 at the Department of Nutritional and Food Sciences, Nutritional Physiology, University of Bonn. After a three-day wash-out period of anthocyanin-free nutrition, the subjects (male/female, age: 20–30) were administered in a fasting state with portions of bilberry-derived food, e.g., juice or puree, standardized on a total anthocyanin content of 400 mg per portion. The urine was collected for 24 h after the food intake, stabilized [47], and stored at −80 °C.

Then, 5 mL of urine was hydrolyzed with two units of β-glucuronidase and 0.5 units of sulfatase for 90 min at 30 °C. After incubation, the samples were acidified with 1.5 mL of formic acid, and 5 µg of the internal standard pelargonidin was added. The samples were homogenized and evaporated at 40 °C under a constant nitrogen stream to a resulting volume of about 500–700 µL. The resulting urine concentrate was filtered through a Chromafil RC 20/15 MS membrane filter from Macherey-Nagel (Düren, Germany) prior to UHPLC-MS analysis.

For method development, anthocyanin-free urine was spiked with either 1 µg/mL or 0.2 µg/mL of each of the five anthocyanidins. The samples were treated as described above; however, as the aglycones were used, no enzymatic hydrolysis was necessary.

#### 3.2.2. Plasma

All five anthocyanins were added in dissolved form to 1 mL of plasma until an amount of 1 or 5 µg of each compound was reached. Subsequently, the plasma was spiked with the equivalent amount of the internal standard pelargonidin-3-*O*-glucoside. Due to the lack of commercially available standards [34], the glucosides were used to substitute the hepatic phase II metabolites. The sample preparation with pepsin-induced plasma proteolysis was performed according to the method established for the analysis of quercetin-3-*O*-glucuronide [20]. Briefly, 42 µL formic acid and 40 µL of a 400 µg/L solution of pepsin in 1 % aqueous formic acid were added. The samples were homogenized and incubated at 37 °C for 20 h. For enzyme inactivation, the 2.5-fold amount of methanol was added. The samples were centrifuged at 17,000× *g*, and the supernatant was evaporated under a constant nitrogen stream at 30 °C to about 500 µL. Human plasma was provided by the University Hospital Bonn, Germany [20].

### 3.3. UHPLC-MS

The UHPLC consisted of an Acquity iClass UPLC Binary Solvent Manager, an Acquity UPLC Sample Manager–FL, and an Acquity UPLC PDA eλ Detector from Waters (Milford, MA, USA) each. The autosampler was cooled at 10 °C, and the column oven was set at 40 °C. For chromatographic separation, an HSS T3 C18 column (2.1 × 150 mm; 1.8 µm) equipped with a VanGuard HSS T3 C18 precolumn (5 × 2.1 mm, 1.7 µm) from Waters (Milford, MA, USA) was used. Eluents were 3% formic acid in water (A) and acetonitrile (B); the flow rate was set at 0.4 mL/min, and the injection volume was 5 µL for all methods. The linear gradient to analyze the anthocyanins was as follows: 0 min 4% B, 7 min 8% B, 13 min 10% B, 19 min 17% B, 23 min 30% B, 23.3 min 100% B, 25.3 min 100% B, and 25.3–25.8 min 4% B. The linear gradient for the analysis of anthocyanidins was 0 min 15% B, 8.5 min 25% B, 9.5 min 100% B, 11 min 100% B, and 11–13 min 15% B.

The MS system was an LTQ XL Iontrap MS^n^ from Thermo Scientific (Waltham, MA, USA) equipped with an electrospray ionization interface operating in positive mode. The spray voltage was 4 kV, and the capillary temperature was set at 325 °C; for the anthocyanidins, the capillary voltage was 9 V with the tube lens voltage at 40 V, and, for the anthocyanins, the capillary voltage was 15 V with the tube lens voltage being 60 V. Consecutive reaction monitoring (CRM) with collision-induced dissociation was used to record the abundance of the analytes, and the respective transitions are shown in Table 2. The instrument was tuned automatically with delphinidin-3-*O*-glucoside for the anthocyanins and pelargonidin for the aglycones. Xcalibur software version 2.2.0.48 from Thermo Fisher Scientific (Waltham, MA, USA) was used.

### 3.4. Validation

The protocols used for the validation were as follows: DIN 32645 for linearity, and limits of detection and quantification; DIN ISO 5725 for recovery and repeatability; ICES CM 1997/E:2 for method precision and repeatability of the instrument, as mentioned in the German Federal Environmental Agency’s validation guideline [35].

The procedures used were described previously in more detail [20]; however, a few minor changes were made. The linearity of the matrix-matched calibration was estimated in the range from 1 ng/mL to 50 µg/mL for both anthocyanins and anthocyanidins. The anthocyanin concentrations in plasma were 1 µg/mL and 5 µg/mL. The anthocyanidin concentrations in urine were 1 µg/mL and 0.2 µg/mL. The solvent for the anthocyanidins was anthocyanidin-free urine, which was concentrated as described above. For anthocyanins, previously proteolyzed plasma was used [20]. The process stability was estimated over a time period of 16 h at a temperature of 10 °C in the sample manager.

### 3.5. Statistics

The statistical calculations were performed using Microsoft Excel and LaborValidate–Labormethoden-Validierung Vers. 2.8. (LABC–Labortechnik Müller und Zilger GbR, Hennef, Germany).

## Figures and Tables

**Table 1:** Validation parameters of anthocyanidins in urine.

	Linearity	Analytical Limits		Precision			Recovery	Repeatability	Robustness
	Range (µg/mL)	LOD (ng/mL)	LOQ (LLOQ) (ng/mL)	System CV (%)	Method CV (%)	(%)		(%)	Process Stability CV (%)
**Dp**	0.125–67	58.1	205.3 (189.7)	9.4	38.0	46.7		51.5	6.7
**Cy**	0.116–71	124.1	427.2 (353.5)	6.8	26.7	78.7		60.5	7.1
**Pt**	0.006–11	12.7	46.0 (15.7)	2.9	15.3	96.5		42.5	7.4
**Pn**	0.005–38	9.6	35.3 (25.6)	2.2	19.1	64.3		31.7	3.7
**Mn**	0.005–15	5.1	18.8 (15.1)	3.7	18.7	65.8		30.9	5.6
**Pg**	0.106–54	120.3	416.2 (412.2)	7.4	--	--		--	--

LOD = limit of detection, LOQ = limit of quantification, LLOQ = lower limit of quantification, CV = coefficient of variation.

**Table 2:** Consecutive reaction monitoring (CRM) transitions of anthocyanins and anthocyanidins.

Analyte	MS (M)^+^	MS^2^ (M)^+^	MS^3^ (M)^+^	Collision Energies MS^2^/MS^3^ (%)
**Dp**	303	257	229	60/45
**Dp-glc**	465	303	-	10
**Cy**	287	213	-	65
**Cy-glc**	449	287	-	10
**Pt**	317	302	274	40/25
**Pt-glc**	479	317	-	10
**Pn**	301	286	258	40/40
**Pn-glc**	463	301	-	10
**Mn**	331	316	299	50/20
**Mn-glc**	493	331	-	10
**Pg**	271	149215	-	67
**Pg-glc**	433	271	-	10

**Table 3:** Results of the analysis of real urine samples.

Urine Sample	c(Dp) (ng/mL)	c(Cy) (ng/mL)	c(Pt) (ng/mL)	Øc(Pt) (ng/mL)	CV (%)	c(Pn) (ng/mL)	Øc(Pn) (ng/mL)	CV (%)	c(Mn) (ng/mL)	Øc(Mn) (ng/mL)	CV (%)
#1	n.d. n.d. n.d. n.d.	n.d. n.q. n.d. n.q.	19.4 20.8 17.5 20.5	19.5	7.6	27.6 34.7 30.3 34.8	31.8	11.1	16.6 22.1 18.8 21.9	19.8	13.3
#2	n.d. n.d. n.d. n.d.	n.q. n.q. n.q. n.q.	22.1 18.8 19.2 22.6	20.7	9.4	37.2 38.4 35.8 38.1	37.4	3.1	26.6 28.7 25.1 22.8	25.8	9.6
#3	n.d. n.d. n.d. n.d.	n.d. n.q. n.d. n.d.	29.7 27.3 25.2 27.2	27.3	6.7	43.2 41.1 40.7 40.1	41.3	3.3	21.6 21.4 19.5 17.4	20.0	9.8
#4	n.d. n.d. n.d. n.d.	n.q. n.q. n.q. n.q.	32.6 30.0 38.0 43.0	35.9	16.1	32.5 29.6 37.0 40.9	35.0	14.2	18.9 19.5 20.0 25.3	21.0	14.1
#5	n.d. n.d. n.d. n.d.	n.d. n.d. n.d. n.d.	26.3 29.8 22.7 26.7	26.4	11.0	45.9 43.9 43.6 44.8	44.5	2.3	29.1 29.2 29.0 29.1	29.1	0.3

n.d. = not detectable, n.q = not quantifiable, c = concentration, Øc = mean concentration, CV = coefficient of variation.

**Table 4:** Validation parameters of anthocyanins in plasma.

	Linearity	Analytical Limits		Precision		Recovery	Repeat-Abilityr	Robust-Ness
	Range (µg/mL)	LOD (ng/mL)	LOQ (LLOQ)(ng/mL)	System CV (%)	Method CV (%)	(%)	(%)	Process Stability CV (%)
**Dp-glc**	0.002–24	2.3	8.1 (4.2)	1.9	15.6	53.8	20.7	5.0
**Cy-glc**	0.003–23	2.0	7.3 (6.9)	2.6	7.7	84.6	10.8	5.7
**Pt-glc**	0.004–25	2.1	7.7 (6.1)	2.8	14.5	68.6	23.2	3.1
**Pn-glc**	0.002–24	1.2	4.1 (3.7)	3.3	4.9	98.8	13.2	5.4
**Mn-glc**	0.002–25	2.2	7.9 (3.3)	5.1	12.2	107.6	20.7	5.5
**Pg-glc**	0.003–25	2.2	8.0 (4.8)	3.0	--	--	--	--

LOD = limit of detection, LOQ = limit of quantification, LLOQ = lower limit of quantification, CV = coefficient of variation.
